# Loading Pressure Induced by 4 mm Implants on the Inferior Alveolar Nerve: A 3D Finite Element Analysis Model

**DOI:** 10.3390/jcm14072535

**Published:** 2025-04-07

**Authors:** Roberta Gasparro, Fabrizio Renno, Simone De Vita, Antonio Lanzotti, Massimo Martorelli, Francesco Penta, Gilberto Sammartino, Pietro Ausiello

**Affiliations:** 1 Department of Neuroscience, Reproductive Science and Dentistry, University of Naples Federico II, 80138 Naples, Italy; roberta.gasparro@unina.it (R.G.); dott.simonedevita@gmail.com (S.D.V.); pietausi@unina.it (P.A.); 2 Department of Industrial Engineering, University of Naples Federico II, 80125 Naples, Italy; fabrizio.renno@unina.it (F.R.); antonio.lanzotti@unina.it (A.L.); massimo.martorelli@unina.it (M.M.); francesco.penta@unina.it (F.P.); 3 CIRMIS, Centro Interdipartimentale di Ricerca in Management Sanitario e Innovazione in Sanità, University of Naples Federico II, 80138 Naples, Italy

**Keywords:** CAD direct modeling, dental implant, finite element analysis, inferior alveolar nerve, nerve injury, short implant

## Abstract

**Background/Objectives**: One of the most serious complications following implant placement in the atrophic posterior mandible is injury to the inferior alveolar nerve (IAN), which can also happen during occlusal loading of the implants. This study investigates the effects of 4 mm implant stress transmission to the inferior alveolar nerve during occlusal loading in cases of severe posterior mandibular atrophy. **Methods**: The computer-aided design (CAD) model was created and modified through Direct Modeling techniques. The structure of cortical and trabecular bones was simplified, and it was modeled as a cylinder block. Finite element analysis (FEA) was carried out in 3D to investigate the pressure distribution over the IAN at different implant-to-nerve distances (1.5 mm, 0.5 mm, and 0.1 mm), and stress and strain deformations were simulated in the mandibular model. **Results**: The results of the pressure analysis on the inferior alveolar nerve indicate that the pressure distribution at different implant-to-nerve distances (1.5 mm, 0.5 mm, and 0.1 mm) is consistently below 0.026 MPa, which corresponds to the maximum pressure range that may block nerve impulses. This occurs even at the theoretical and simulated distance of 0.1 mm, suggesting that cortical bone stiffness plays a crucial role in mitigating stress at reduced implant-to-nerve proximities. **Conclusions**: Within the limits of this study, ultra-short implants can be placed even less than 0.5 mm (up to 0.1 mm under the 3D-FEA hypothesis) above the inferior alveolar nerve under the 3D-FEA hypothesis, while maintaining pressure below the threshold value. This is due to the rigidity of the cortical bone, which helps to reduce pressure transmission to the nerve. These findings may expand the indications for ultra-short implants, even in mandibles with a residual bone height of just 4 mm.

## 1. Introduction

The implant-supported fixed rehabilitation of the human mandible with vertical bone atrophy in the posterior area represents a common clinical challenge [1]. When sufficient bone width exists but vertical height is inadequate, various clinical solutions can be employed by different surgical methodologies, such as onlay and inlay bone grafts, guided bone regeneration (GBR), inferior alveolar nerve (IAN) transposition, and the use of angulated implants placed using modern navigation or guided surgery systems [2,3,4]. However, these approaches may involve higher complication rates, increased morbidity, longer healing times, or require advanced surgical tools and experience [5,6]. In contrast, ultra-short implants offer a minimally invasive solution, particularly in patients who are unwilling or unable to undergo complex grafting procedures or nerve transposition [7]. According to the ITI Consensus Report, short implants are defined as implants of less than 6 mm in length, while ultra-short implants are defined as 4 mm-long implants [8]. Short implants are increasingly favored as alternatives to vertical bone augmentation surgeries [9,10] due to their reduced complication rates, shorter rehabilitation times, and improved patient satisfaction [11,12]. In the posterior mandible, bone atrophy is commonly encountered in edentulous patients. This anatomical situation is favorable for ultra-short dental titanium implants, as other surgical techniques can be less predictable due to biological and anatomical challenges: the proximity of the inferior alveolar nerve, the reduced blood supply, and the presence of thin, soft tissue [13,14].

One of the most serious complications following implant placement in this area is injury to the inferior alveolar nerve (IAN), with reported incidence rates ranging from 0% to 40% [15,16,17,18]. It can often occur during implant placement. Less frequently, it can happen during the occlusal loading of the implants [19,20]. To minimize such risks, 3D assessments of the mandibular canal using X-ray tomography, the selection of a proper implant length, and proper bone drilling and implant positioning are crucial [21].

Previous studies have clinically investigated the ideal distance between standard implants and the mandibular canal nerve to ensure both implant integrity and nerve function [19,20]. A recommended canal distance of 1.5 mm has been suggested to prevent excessive pressure on the IAN and the associated sensory disturbances from occlusal forces transmitted through the implant [19]. In cases where multiple implants are connected, a minimum distance of 1.0 mm is recommended to avoid nerve damage [20].

However, in cases of severe posterior mandibular atrophy, where patients are unwilling to undergo invasive bone reconstruction techniques, achieving a 1.5 mm safety distance can be challenging, even for 4 mm implants. Consequently, these implants are sometimes placed with less than the recommended safety distance, utilizing the total residual alveolar ridge height [19,20]. There are currently no in vivo or in vitro studies showing the anatomical or functional consequences of this in terms of strain and stress compression, or the deformation of 4 mm implants in the mandibular bone close to the lower alveolar nerve. This study aimed to fill that gap by simulating the effects of occlusal loading on a 4 mm dental implant in atrophic bone using 3D finite element linear analysis. Finite element analysis (FEA) is a well-described computational method able to simulate physical and biological phenomena in silico when a detailed 3D computer-aided design (CAD) model of dental tissues or bone has been aided by complex scanner technology [22,23,24,25,26,27,28]. The study tested the clinical assumption that the occlusal loading of a 4 mm implant placed close to the inferior alveolar nerve does not generate nerve pressure exceeding the physiological threshold. The goal of this investigation was to evaluate the stress and strain deformation of the cortical and trabecular bones of 4 mm implants under occlusal loading, and the pressure distribution on the IAN at varying implant-to-nerve distances. To the best of our knowledge, no previous FEA studies have assessed nerve pressure thresholds for ultra-short implants placed in such close proximity to the nerve, especially using a CT-calibrated model that accounts for variations in bone stiffness.

## 2. Materials and Methods

**Figure 1:**
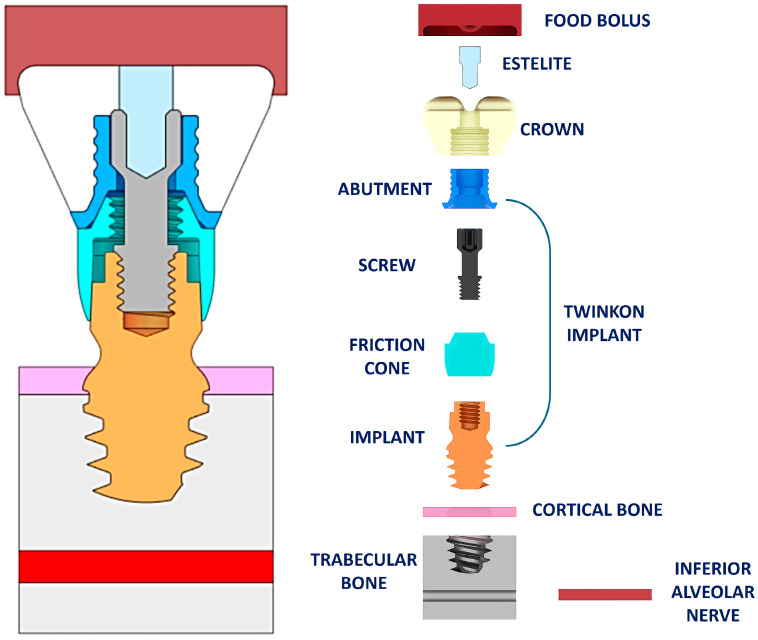
CAD model of the case studied. On the left the assembly is shown, on the right the exploded view of the complete CAD model is depicted.

A static structural FEM analysis was performed to determine the pressure of the model depicted in Figure 1, involving the interface between the nerve and mandibular canal, and, especially, to estimate the pressure value on the inferior alveolar nerve. The CAD model was created and modified through Direct Modeling techniques (ANSYS SPACECLAIM^®^ software, ANSYS 23.1, ANSYS Inc., Houston, TX, USA). Starting from the step file of the 4 mm implant (twinKon^®^ implant, GlobalD, Brignais, France), it was completed by adding the food bolus, prosthetic crown, cortical and trabecular bones, and the inferior alveolar nerve for a more realistic analysis. The structure of the cortical and trabecular bones was simplified, and it was modeled as a cylinder block (8 mm diameter and 8 mm length), as in [29]. The robustness of the model to this assumption was empirically confirmed.

**Figure 2:**
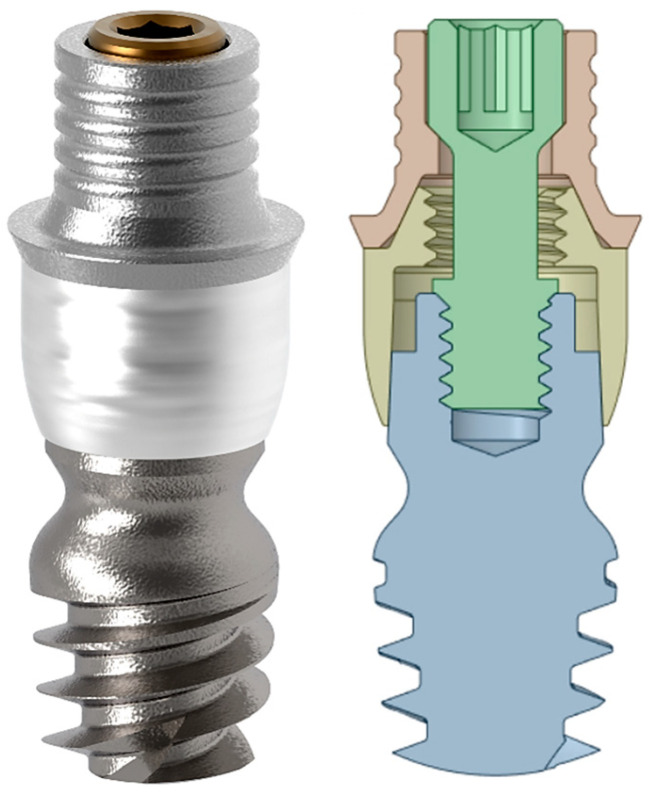
On the left, the rendered view of the 4 mm twinKon^®^ implant used as case study is shown, on the right, there is the related section view.

Figure 1 shows the complete CAD model and its exploded view, whereas Figure 2 shows the rendered image of the 4 mm twinKon^®^ implant.

**Figure 3:**
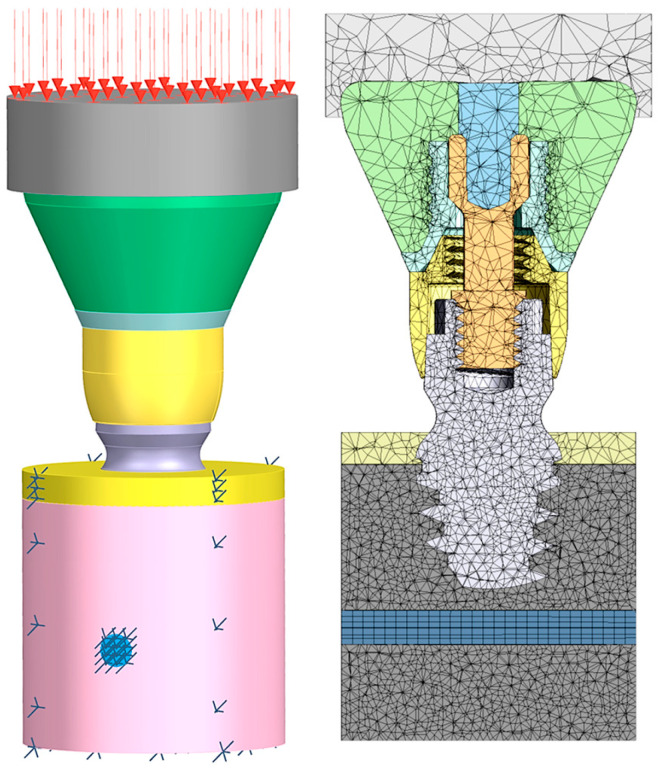
Boundary conditions used (**left**): 200 N uniform pressure on the top surface of the bolus and fixed constraints imposed on the bottom face of the trabecular bone, the lateral faces of the cortical and trabecular bones, and the inferior alveolar nerve. Mesh characteristics (**right**): tetrahedral meshes with different element dimensions for each component were used. In some specific cases, the dimensions of the mesh elements of the trabecular bone, cortical bone, mandibular canal, and inferior alveolar nerve were intentionally chosen to be less than 0.1 mm.

The analysis was carried out by employing the ANSYS FEA^®^ software (ANSYS 23.1, ANSYS Inc., Houston, TX, USA). Fixed constraints were imposed on the bottom face of the trabecular bone, on the lateral faces of the cortical and trabecular bones, and on the inferior alveolar nerve. A 200 N vertical load was applied to the top surface of the bolus as uniform pressure (Figure 3). Each vector shown in Figure 3 represents a proportion of the total load, so the sum of all the vectors equals 200 N.

To simplify and speed up analyses, tetrahedral meshes with different element dimensions for each component were used. To do so, the FEM model was initially characterized by 0.5 mm element meshes with 368,393 nodes and 247,251 elements. Afterward, in some specific cases, the dimensions of the mesh elements of the trabecular bone, the cortical bone, the mandibular canal, and the inferior alveolar nerve were intentionally chosen to be less than 0.1 mm. The ANSYS FEA^®^ software’s automatic adaptive meshing functionality was used, and convergence was considered to be achieved when relative changes of less than 10% were observed in stress, strain, and contact pressure at the interfaces between the implant, inferior alveolar nerve, and trabecular bone. In the present simulation, due to the likely uncertainties associated with CT imaging, the simplified isotropy hypothesis was adopted for the sake of simplicity, and more refined mechanical modeling was avoided. Thus, to simplify the focus on implant geometry and loading conditions and avoid the added complexity and uncertainty of anisotropic properties, homogeneous isotropic linear elastic behavior was considered for all the solid elements. Future works will investigate more detailed anisotropic modeling of bones and more refined boundary conditions to improve the predictive accuracy of the model. The Young’s moduli and Poisson’s ratios used for the analysis are provided in Table 1. As in a previous study by the present authors, the inferior alveolar nerve was modeled as isotropic too [8], with Young’s modulus E = 1.3 MPa and Poisson ratio ν = 0.4.

**Figure 4:**
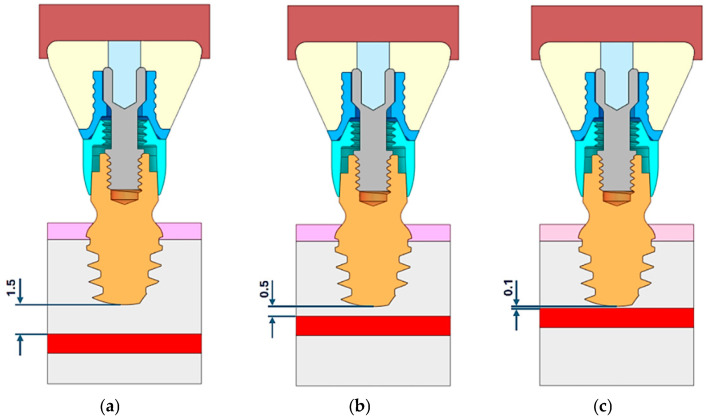
Distance between the fixture bottom and the upper part of the mandibular canal. Three models considering different distances between the bottom part of the implant and the upper part of the mandibular canal were created, (**a**) d = 1.5 mm, (**b**) d = 0.5 mm, (**c**) d = 0.1 mm.

For the study of stress and strain, one reference case was analyzed based on a bone density value of 700 Hounsfield Units (HU), which corresponds to a Young’s modulus (E) of 4.5 GPa. The pressure distribution on the inferior alveolar nerve was analyzed to determine how the distance between the implant and the mandibular nerve can influence the nerve’s integrity and its physiological activity. Three models considering different distances between the bottom part of the implant and the upper part of the mandibular canal were created: (a) d = 1.5 mm, (b) d = 0.5 mm, and (c) d = 0.1 mm (Figure 4). For all the simulations, an orthogonal canal vertical to the axis of the implant was considered.

To define the Young’s modulus of the trabecular bone to be used in the FEM analysis, an experimental procedure was adopted. After the mesh definition, it is necessary to assign mechanical properties to the various parts of the model. They are derived from the computed tomography (CT) data of 100 patients with an atrophic posterior mandible, with a height of up to 5 mm. Then, according to [30], the material properties, based on the density of the bone tissue at its specific location, are assigned to each element of the mesh. These variable material properties have to be mapped onto the finite element (FE) models utilizing the Bonemat^®^ software V3.1, (Istituto Ortopedico Rizzoli, Bologna, Italy) [31,32,33]. Then, the local elastic modulus based on the radiographic density (ρQCT) measured in the computed tomography (CT) scan was calculated. This elastic modulus is derived from the apparent bone density (ρAPP) at the element’s location, as indicated in Equation (3) [30]. The apparent density is calculated by considering all the points from the CT grid that fall within each element of the model. So, for the case studied, the radiographic density (ρQCT) and the apparent bone density (ρAPP = ρASH/0.6) according to [32] are given using the following equation:

ρQCT = −0.016404 + 0.00085164 HU(1)

ρASH = 0.079 + 0.877 ρQCT(2)

with ρ expressed in g/cm^3^, E in MPa, and a Poisson’s ratio equal to 0.3.

Bone density is directly related to its elasticity. The elastic modulus E is influenced by density at specific anatomical sites, and may be expressed according to [34] as follows:

E = 4730ρ_APP^1.56(3)

So, the relationship between ash density (ρASH) and apparent density (ρAPP) was determined as follows:

E = 10,494 ρ_ASH^1.56(4)

Therefore, it was possible to calculate the Young’s modulus as a function of the Hounsfield Unit by means of Equation (4). The fourteen Young’s moduli used to study the pressure variation as a function of bone density changes are presented in Table 2.

Pressure was then determined for all models (a, b, and c), and for each of the fourteen previously calculated Young’s modulus values. The literature data suggest that pressures ranging from 100 to 200 mmHg (i.e., 0.013 to 0.026 MPa) applied for 30 to 60 s to the inferior alveolar nerve may lead to a block of the nervous impulse [35], so a pressure inferior to 0.026 MPa was considered our threshold. Since nerve pressure is expected to increase as bone density decreases, the main target of this analysis was to determine whether trabecular bone atrophy can limit the effect of the loads on the underlying jaw structures.

## 3. Results

### 3.1. Stress and Strain Deformation

**Figure 5:**
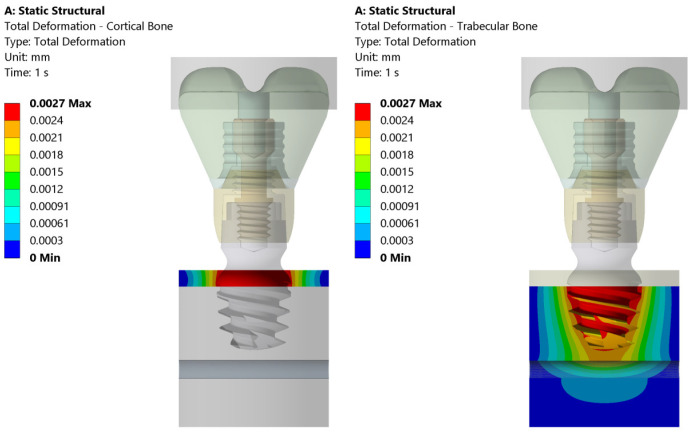
Total deformation in cortical and trabecular bones under the 200 N vertical load applied uniformly to the bolus. The color scale indicates the magnitude of strain, with the maximum strain observed both in the cortical zone and at the implant thread.

**Figure 6:**
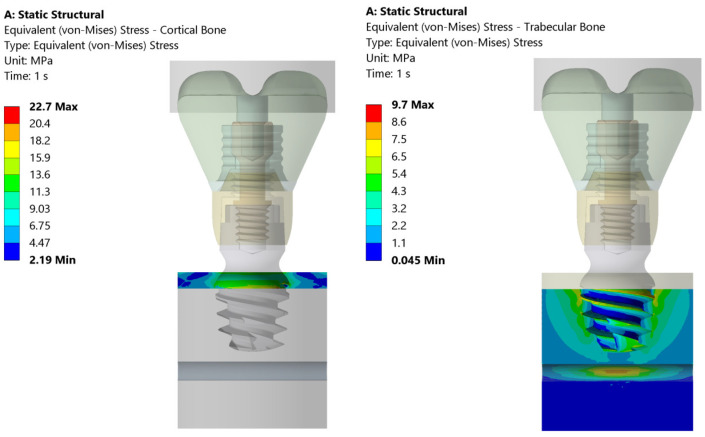
Distribution of equivalent stresses in cortical (**left**) and trabecular bone (**right**) subjected to the 200 N vertical load. The color map represents the value of the equivalent stress (von Mises) (in MPa) and highlights the higher stress concentration in the region of cortical bone near the implant interface compared to the more evenly distributed stress in trabecular bone.

**Figure 7:**
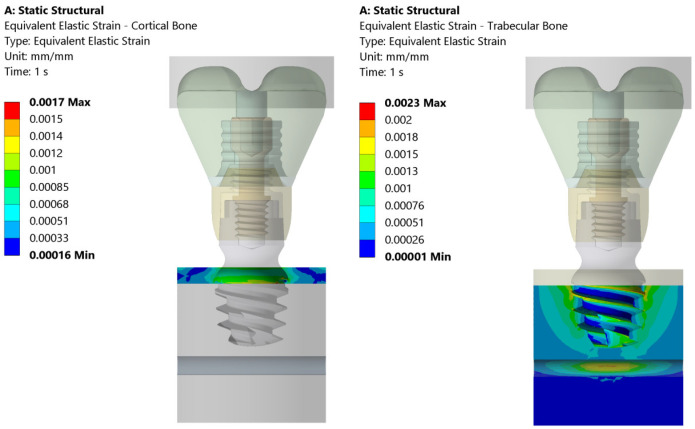
This figure illustrates the distribution of equivalent elastic strain under the 200 N vertical loading condition in both the cortical and trabecular bone regions. Similar strain concentrations are observed across both the cortical and trabecular bone regions. The boundary conditions were applied so that the lateral surfaces were fixed, ensuring that the observed strain patterns were primarily due to the applied load and material properties.

The total deformation, von Mises equivalent stress, and equivalent strain are shown in Figure 5, Figure 6 and Figure 7. On the left side, the deformation of the cortical bone is reported; on the right side, the deformation of the trabecular bone. Figure 5 shows that the maximum deformation (red) is seen near the top cortical region, in direct contact with the implant, whereas the surrounding areas display a gradual decrease in deformation intensity. On the contrary, the trabecular bone exhibits more widespread deformation, with a concentration of high stress (red/yellow) at the implant threads and deeper into the bone structure.

Figure 6 illustrates the equivalent (von Mises) stress distribution in both the cortical and trabecular bones under static loading conditions. The cortical bone experiences a maximum stress of 22.7 MPa, with higher stress concentrations localized in the upper region of the implant–bone interface. In contrast, the trabecular bone exhibits a maximum stress of 9.7 MPa, distributed primarily around the implant threads and deeper bone layers. The stress values progressively decrease outward, indicating that the trabecular bone absorbs more load over a wider area compared to the cortical bone.

Figure 7 presents the equivalent elastic strain distribution in the cortical and trabecular bones. The cortical bone exhibits a maximum strain of 0.0017, concentrated at the upper implant–bone interface. The strain values decrease outward, indicating that the cortical bone, due to its high stiffness, restricts deformation to a localized region. In contrast, the trabecular bone shows a maximum strain of 0.0023, primarily around the implant threads and extending deeper into the bone.

### 3.2. Pressure Distribution

**Figure 8:**
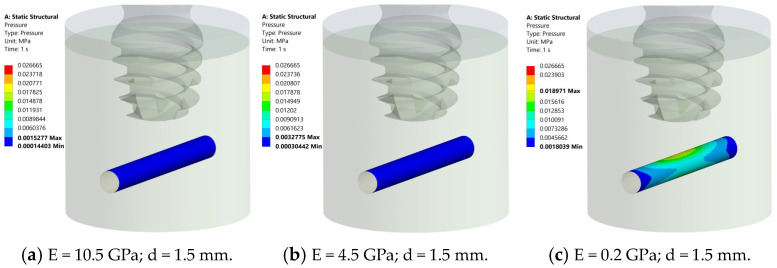
Results of pressure distribution across nerve structure at d = 1.5 mm from implant for different bone elastic moduli. (**a**) E = 10.5 GPa, nerve is deep blue, suggesting 0.001 MPa max pressure. (**b**) E = 4.5 GPa, comparable blue region, 0.003 MPa max pressure. (**c**) E = 0.2 GPa, nerve changes color from blue to green and yellow, 0.018 MPa max pressure. Pressure consistently below 0.026 MPa, confirming 1.5 mm gap safety.

**Figure 9:**
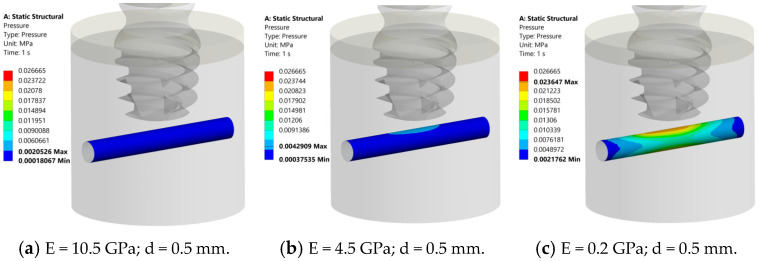
Results of the pressure analysis on the inferior alveolar nerve (d = 0.5 mm). (**a**) 10.5 GPa: nerve appears uniformly deep blue (max pressure 0.002 MPa). (**b**) 4.5 GPa: noticeable gradient in central nerve (blue to azure) at max pressure of 0.004 MPa. (**c**) 0.2 GPa: pressure gradient (blue to green, yellow, and red) at max pressure of 0.023 MPa. In all cases, peak pressure is below 0.026 MPa (safe).

**Figure 10:**
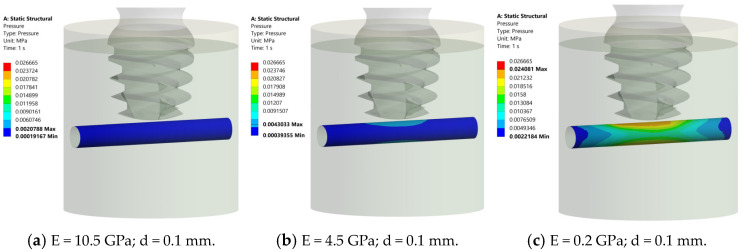
Pressure distribution in the nerve structure at different elastic moduli. (**a**) E = 10.5 GPa, maximum pressure = 0.002 MPa, deep blue. (**b**) E = 4.5 GPa, pressure gradient from blue (0.004 MPa) to azure, central region. (**c**) E = 0.2 GPa, transition from blue to green, yellow, and red (0.024 MPa), central nerve region. All cases confirm pressures below 0.026 MPa, confirming the safety of a 0.1 mm distance.

The summary of the results of the pressure distribution for three cases (E = 0.2 GPa, E = 4.5 GPa, and E = 10.5 GPa) are shown in Table 3 and in Figure 8, Figure 9 and Figure 10.

At 1.5 mm between the implant and the nerve, the nerve structure appears deep blue for E = 10.5 GPa and 4.5 GPa (Figure 8a,b), suggesting that it experiences very low pressure (maximum pressure of 0.001 MPa and 0.003 MPa, respectively). At E = 0.2 GPa, the nerve structure in the middle exhibits a gradient of pressure, transitioning from blue to green and yellow (maximum pressure 0.018 MPa) (Figure 8c). In all cases, the pressure was always inferior to 0.026 MPa, so this distance can be considered “safe”.

At 0.5 mm between the implant and nerve, the nerve structure appears deep blue for E = 10.5 GPa (maximum pressure of 0.002 MPa) (Figure 9a). At E= 4.5 GPa, the nerve structure in the middle exhibits a gradient of pressure, transitioning from blue to azure (maximum pressure (0.004 MPa), (Figure 9b). At E = 0.2 GPa, the nerve structure in the middle exhibits a gradient of pressure, transitioning from blue to green, yellow, and red (maximum pressure 0.023 MPa) (Figure 9c). In all cases, the pressure was always inferior to 0.026 MPa, so this distance can also be considered “safe”.

At 0.1 mm between the implant and nerve, the nerve structure appears deep blue for E = 10.5 GPa (maximum pressure of 0.002 MPa) (Figure 10a). At E= 4.5 GPa, the nerve structure in the middle exhibits a gradient of pressure, transitioning from blue to azure (maximum pressure (0.004 MPa), (Figure 10b). At E = 0.2 GPa, the nerve structure in the middle exhibits a gradient of pressure, transitioning from blue to green, yellow, and red (maximum pressure 0.024 MPa) (Figure 10c). In all cases, the pressure was always inferior to 0.026 MPa, so this distance can also be considered “safe”.

**Figure 11:**
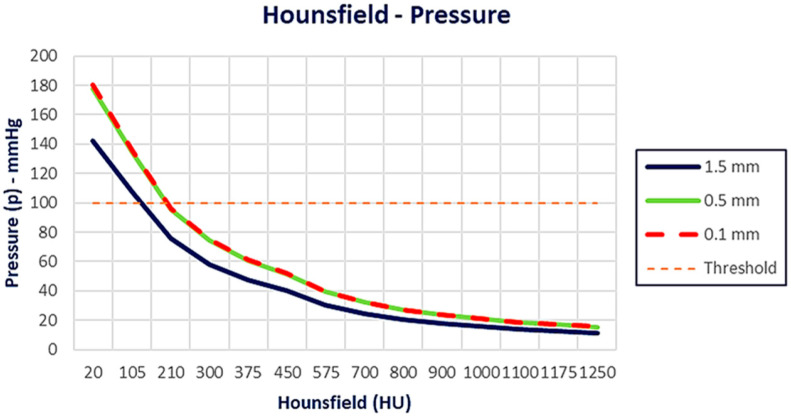
Pressure distribution as a function of Hounsfield. As Hounsfield increases, pressure decreases for all different distances. The 0.1 mm and 0.5 mm cases follow a very similar trend, while the 1.5 mm case shows slightly lower pressure values overall.

**Figure 12:**
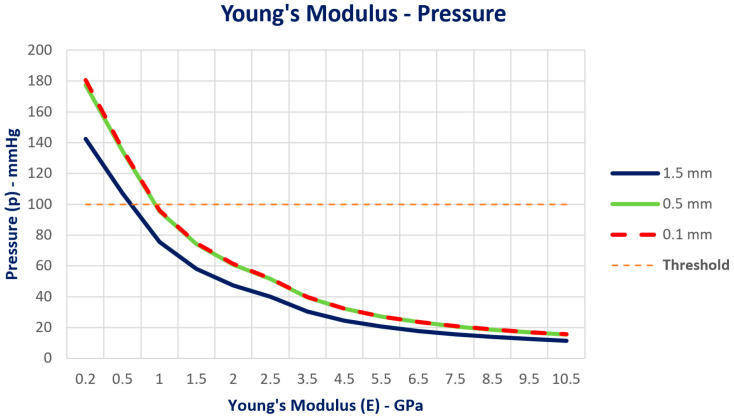
Pressure distribution as a function of Young’s Modulus. As Young’s Modulus increases, pressure decreases for all different distances. The 0.1 mm and 0.5 mm cases follow a very similar trend, while the 1.5 mm case shows slightly lower pressure values overall.

After the evaluation of the pressure distribution as a function of bone density, the following graphs (Figure 11 and Figure 12) were generated, illustrating the pressure (mmHg) as a function of the HU values and Young’s modulus, respectively. At lower HU values (from 100 to 200 HUs), the pressure values are significantly higher, above the threshold line for all distances. As Hounsfield increases, the pressure decreases for all different distances. The 0.1 mm and 0.5 mm cases follow a very similar trend, while the 1.5 mm case shows slightly lower pressure values overall (Figure 11).

The same behavior was achieved based on Young’s Modulus. At lower Young’s Modulus values (from 0.5 to 1 GPa), the pressure values are significantly higher, above the threshold line for all distances. As Young’s Modulus increases, the pressure decreases for all different distances. The 0.1 mm and 0.5 mm cases follow a very similar trend, while the 1.5 mm case shows slightly lower pressure values overall (Figure 12).

## 4. Discussion

The aim of this investigation was to evaluate the stress and strain deformation of the cortical and trabecular bones with 4 mm implants under occlusal loading and the pressure distribution on the IAN at different distances. The findings of the von Mises equivalent stress distribution indicated that the trabecular bone absorbs more load over a wider area compared to the cortical bone. This difference highlights the varying biomechanical behaviors of these bone types, influencing implant stability and long-term success [36]. Moreover, the equivalent elastic strain maps indicated that cortical bone, due to its high stiffness, restricts deformation to a localized region, emphasizing the importance of implant positioning and bone quality in stress and strain distribution, impacting implant longevity and stability [37]. Numerous analyses were carried out to calculate pressure as a function of bone density [38,39,40,41]. The fourteen grades (HU and Young’s modulus), previously determined and shown in Table 2, were considered, as well as the distance d between the bottom of the implant and the top of the mandibular canal (a = 1.5 mm, b = 0.5 mm, and c = 0.1 mm models). The results indicate that pressures approaching the 200 mm Hg threshold are observed only for very low HU values (<200), and consequently for a low Young’s modulus (<1 GPa), though they remain below this limit. This also occurs in the case of model c, as shown in Figure 10, where the distance d is 0.1 mm. In all other cases, the pressure values are consistently low and far from the considered threshold. This behavior is illustrated in Figure 8b,c and Figure 9b,c, which show the pressure trend as the HU and Young’s modulus vary. In these figures, the pressure values calculated for models b and c are very similar, and for this reason, the two corresponding curves are nearly overlapping. The minimal pressure variation at the 0.5 mm and 0.1 mm distances may be due to the different rigidity values of the bone (E), or to its limited ability to transmit additional stress, in addition to the non-linear deformation behavior and stress saturation effects analyzed in [39]. In fact, when the implant is far enough from the nerve (i.e., 1.5 mm), as shown in Figure 8, stress is spread more effectively through the trabecular and cortical bones, generating very low pressure on the nerve, independently from the E modulus considered (10.5 GPa, 4.5 GPa, 0.2 GPa). As the distance decreases (0.5 mm and 0.1 mm), a lower bone thickness is available to absorb and distribute stress, but it begins to have a more rigid behavior, limiting the increase in pressure on the nerve [19,20]. This behavior depends on the characteristics of bone resorption in the posterior mandible, with a complete reduction in the trabecular bone and an increase in the corticalization, which leads to major rigidity [42,43]. Therefore, as the distance decreases, the ability of the bone to deform and transmit additional pressure to the nerve also decreases, which explains the minimal pressure variation between 0.5 mm and 0.1 mm.

In summary, this characteristic may contribute to the bone’s ability to provide protective buffering for nerves within a critical threshold distance. As such, the findings suggest that when implants are positioned closer than 1.5 mm, the bone may absorb in a self-limiting process, where stress propagation diminishes as it reaches the nerve boundary, likely due to a combination of the material’s rigidity and inherent stress-shielding properties. According to the present investigation, a previous FEA study [18] was used to evaluate stress distribution and deformation on surrounding peri-implant bone and neural integrity. While our study suggests that implant distance from the nerve should be maximized within the safe limits (with short implants), the referenced study provides additional guidance on achieving this via tilt angles when direct distancing is constrained by bone height limitations [19]. Gümrükçü et el. [20] examined the biomechanical behavior of three-element fixed partial dentures supported by two osseo-integrated implants and concluded that dental implants placed using the inferior alveolar nerve lateralization technique pose a lower risk of bone loss compared to short implants under similar conditions. This difference highlights a key distinction from the present study, which focuses on a single-unit implant crown rather than a bridge, underscoring the limitations in generalizing these findings to all clinical scenarios. Our findings support previous finite element analyses, reinforcing the idea that implant positioning and structural support are essential in managing bone stress and minimizing resorption [23]. However, it is worth noting that the authors emphasized an additional factor: the height of the prosthesis and the resulting lever effect in cases of atrophic bones, which can intensify stress around the implant and potentially accelerate bone loss [22]. This consideration suggests that beyond implant positioning, the structural characteristics of the prosthesis itself play a significant role in the biomechanical outcomes, particularly in compromised support conditions [43,44]. The findings of Sammartino et al. [18] closely align with our research focus on stress transmission to the inferior alveolar nerve during implant placement. Using a numerical model and boundary element method, the study evaluates the occlusal stress transmitted to the nerve at various implant-to-canal distances, emphasizing a recommended minimal distance of 1.5 mm to prevent nerve injury under functional loads. This reinforces findings from our own study, where stress on the IAN is shown to increase as implant-to-nerve distance decreases, highlighting a safe threshold distance for implant positioning to minimize nerve pressure and potential neuropathy. One notable difference, however, lies in the biomechanical modeling approach. Sammartino’s study [19] leverages the boundary element method, which is adept at linear analysis and managing complex implant-threaded geometries, whereas our study utilizes finite element analysis, which is advantageous for capturing non-linear tissue behaviors, such as the bone’s limited ability to deform under high stress. Despite these methodological differences, both studies converge on the conclusion that nerve preservation requires the careful planning of implant placement, particularly when distances approach 1 mm. The pressure threshold of 0.026 MPa corresponds to the literature-based values (100–200 mmHg), beyond which temporary or permanent nerve conduction blocks may occur [35]. Although this is a simplification, it provides a clinically relevant benchmark for evaluating the risk of inferior alveolar nerve injury. Our simulation showed that even at the minimal distance of 0.1 mm, the pressure values remained below this critical threshold across a wide range of bone densities. These findings suggest that, under ideal conditions, ultra-short implants may be positioned with minimal nerve clearance without exceeding the physiologically tolerable pressure levels, supporting their use in anatomically challenging cases. The present study also showed how pressure varies in smaller increments as the implant approaches the nerve, particularly between 0.1 mm and 1.5 mm distances. This close-range analysis demonstrates that the cortical bone around implants behaves increasingly rigidly as distance decreases, ultimately limiting pressure increases on the nerve at very close proximities.

Although a physiological bone loss of 0.2 mm per year may seem significant for ultra-short implants, clinical studies have shown that, with proper implant design and load management, these implants can maintain stable marginal bone levels over time. This suggests that bone remodeling patterns may differ from theoretical predictions, probably for higher bone densities of the atrophic mandible [45,46,47,48].

In the present investigation, several limitations should be noted. First, this study utilizes a finite element model that assumes idealized isotropic material properties, which may not fully capture the anisotropic nature of bone and nerve tissues in vivo. Additionally, the FEA model uses simplified assumptions that may not accurately replicate in vivo behavior. While the FEA model simplifies cortical and trabecular structures, variations in bone density and quality across different patient populations may affect stress distribution, and should be considered in clinical applications [19,20,21]. Lastly, this study lacks clinical validation and assesses static occlusal loading; dynamic or repetitive loading conditions might produce different stress patterns and should be examined in future research to more accurately reflect real-life functional loading. Although this study employed a standardized, fixed height for the alveolar canal and did not assess variations in the vertical position of the inferior alveolar nerve, we acknowledge that such anatomical variability may significantly impact implant planning and risk assessment. A major strength of this study lies in the use of a detailed, patient-derived 3D finite element model to simulate clinically relevant implant-to-nerve distances under high bone densities. However, as this was an in silico analysis based on idealized conditions, further in vitro and clinical studies are necessary before the results can be generalized to all clinical scenarios, and incorporating different alveolar canal heights could yield more clinically relevant insights and contribute to the refinement of surgical safety protocols.

## 5. Conclusions

Within the limitations of this in silico study, our 3D finite element analysis suggests that 4 mm ultra-short implants placed as close as 0.1 mm from the inferior alveolar nerve may not exceed the critical pressure threshold for nerve function, particularly due to the protective rigidity of the cortical bone. However, this model is based on idealized and simplified material properties, assumes linear elastic and isotropic behavior, and does not account for dynamic occlusal forces or biological variability. Additionally, the findings have not yet been validated in clinical or experimental settings. Future research should include in vitro experiments and clinical trials to confirm these biomechanical results, assess long-term outcomes, and evaluate the safety and predictability of ultra-short implants in anatomically limited sites.

## Figures and Tables

**Table 1:** Young’s modulus and Poisson’s ratio used for the FEM analyses.

	Structure	Young’s Modulus	Poisson’s Ratio
1	Bolus	3.4 GPa	0.10
2	Cortical Bone	13.7 GPa	0.30
3	Trabecular Bone	4.5 GPa	0.30
4	Estelite P-Block (Composite Resin)	13.8 GPa	0.30
5	Ti-6al-4v	113 GPa	0.35
6	Inferior Alveolar Nerve	0.0013 GPa	0.40
7	Zirconia (Crown)	200 GPa	0.30

**Table 2:** Young’s Modulus calculated as a function of HU values.

	HU	ρ_QCT_	ρ_ASH_	E (GPa)
1	1250	1.048	0.998	10.5
2	1175	0.984	0.942	9.5
3	1100	0.920	0.886	8.5
4	1000	0.835	0.812	7.5
5	900	0.750	0.737	6.5
6	800	0.665	0.662	5.5
7	700	0.580	0.587	4.5
8	575	0.473	0.494	3.5
9	450	0.367	0.401	2.5
10	375	0.303	0.345	2.0
11	300	0.239	0.289	1.5
12	210	0.162	0.221	1.0
13	105	0.073	0.143	0.5
14	20	0.001	0.080	0.2

**Table 3:** Pressure distribution according to Young’s modulus and distances.

	1.5 mm	0.5 mm	0.1 mm
**E = 0.2 GPa**	p: 0.018 MPa	p: 0.023 MPa	p: 0.024 MPa
**E = 4.5 GPa**	p: 0.003 MPa	p: 0.004 MPa	p: 0.004 MPa
**E = 10.5 GPa**	p: 0.001 MPa	p: 0.002 MPa	p: 0.002 MPa

E: Young’s modulus; p: pressure.

## Data Availability

Data are contained within the article.
